# Identification of the Radiative Parameters-Albedo and Optical Thickness—Of the *Juncus maritimus* Fiber

**DOI:** 10.3390/ma16051891

**Published:** 2023-02-24

**Authors:** Marcelo Borges dos Santos, Luís Mauro Moura, Dominique Baillis

**Affiliations:** 1 Thermal System Laboratory (LST), Graduation Program in Mechanical Engineering, Pontificia Universidade Católica do Paraná, Curitiba 80910-215, Brazil; 2 Sesi Institute of Innovation, Federation of Industries of the State of Parana ISI-FIEP, Curitiba 80215-090, Brazil; 3 Univ Lyon, INSA Lyon, LaMCoS, CNRS UMR5259, LaMCoS, 69621 Villeurbanne, France

**Keywords:** *Juncus maritimus*, albedo, optical thickness, RTE, discrete ordinate method, Gauss linearization, Neumann method

## Abstract

The present work aims to characterize the radiative thermal properties albedo and optical thickness of *Juncus maritimus* fibers using a FTIR spectrometer. Measurements of normal/directional transmittance and normal and hemispherical reflectance are performed. The numerical determination of the radiative properties is conducted through the computational treatment of the Radiative Transfer Equation (RTE) using the Discrete Ordinate Method (DOM), together with the inverse method, which is done through Gauss linearization. As it is a non-linear system, iterative calculations are necessary, which demand a significant computational cost, and, to optimize this problem, the Neumann method is used for the numerical determination of the parameters. These radiative properties are useful to quantify the radiative effective conductivity.

## 1. Introduction

**Figure 1:**
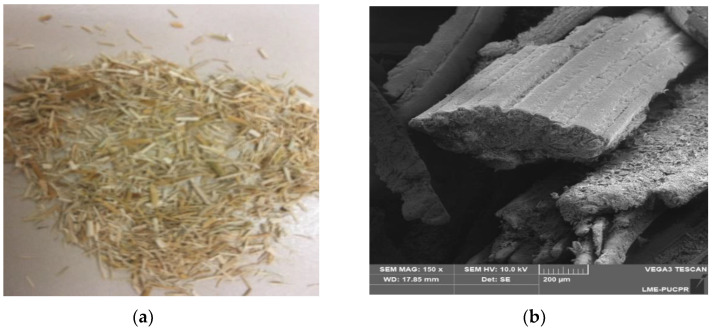
Physical aspects of juncus fiber (**a**) and its microscopic image (**b**).

*Juncus maritimus* fiber, the object of analysis in this work, is a biodegradable, ecologically sustainable material that can be obtained continuously throughout the year. The *Juncus maritimus* is a plant that is found in countries that have a high rate of solar incidence, so it is interesting to analyze the radiative properties of the fiber, as a function of solar radiation and the spectrum band belonging to infrared [1]. Some studies on *Juncus maritimus* fiber have already been conducted, and some properties have already been previously determined, such as the thermal conductivity of *Juncus maritimus* fiber, which is relatively low, ranging from 0.521 ± 0.0036 to 0.171 + 0.00223 (W·m^−1^K^−1^), depending on the fiber porosity degree [2]. *Juncus maritimus* fiber mixed with cement presents a desirable increase in mechanical properties in terms of strength [3,4]. Figure 1a,b show, respectively, a fiber sample used in the radiative property analysis and a microscopic view of the fiber. The thermal conductivity together with the absorption of thermal energy in the form of infrared radiation allows for a more complete thermal characterization of the *Juncus maritimus* fiber, since the heat transfer by radiation inside the porous material can significantly contribute to the total heat transfer measured due to the high porosity (70%). For traditional porous insulating media used in construction applications (such as polystyrene foam at room temperature), the radiation contribution can reach 35% of the total effective thermal conductivity. However, radiation contribution was still neglected in previous works dealing with thermal conductivity for bio-based materials such as *Juncus maritimus* fibers [3]. Thus, it is important to characterize radiative conductivity of such media. As expected, the gain in terms of thermal insulation, together with the increase in mechanical strength, justifies the wide use of this material.

Some studies have been conducted regarding the behavior of the fiber in relation to the energy absorption in the form of radiation [5], but the RTE with spectrum analyses has a broader application, as it analyzes the medium contribution to the dispersion of radiation, allowing to determine a larger number of parameters. In addition, the determination of the parameters was conducted through the discretization of the RTE (Radiative Transfer Equation) and the Gaussian linearization method. These are consolidated mathematical methods and have already been successfully applied, for instance, in the determination of thermo-radiative properties in cellulose-based materials [6]. It is important to mention that the Gaussian linearization method, which consists of determining the solution of a linear system recursively until the problem converges, can represent a significant computational cost, and, to circumvent this problem, this article presents the Neumann method, which has satisfactory results, for instance, in the determination of heat diffusivity [7].

## 2. Experimental Design

**Figure 2:**
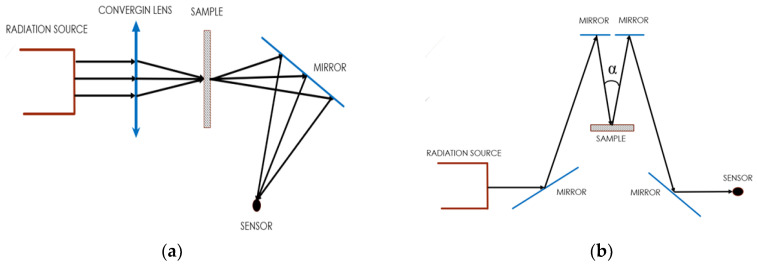
Diagram of the path of the radiative beam over the sample referring to the transmittance (**a**) and normal reflectance measurements, respectively (**b**).

The experiment was conducted using 100 FT-IR and FT-NIR spectrometer devices, which operate in the infrared band with a wavelength ranging from 1.2 to 15 µm, with the maximum intensity corresponding to a wavelength of 4.26 µm, which indicates that the source temperature of the spectrometer is approximately 680.29 K, obtained by the Wien displacement law [8]. The air inside the equipment was previously purged, eliminating water vapor and CO_2_ for all experiments performed. Transmittance measurements were done with the sample positioned at the focus of the spectrometer converging lens. The radiative beam falls directly on the surface of the sample and passes through it. The numerical value of the unabsorbed energy intensity of the beam that passes through the sample was determined by the sensor inside the spectrometer. According to the 100 FT-IR and FT-NIR spectrometer manual, the incident beam opening angle is 5°. In order to obtain the radiative properties dependent on normal reflection, the accessory already specified for the reflectivity test was coupled to the spectrometer. The incident radiative beam on the surface of the sample was at an angle, α, of approximately 10°. The fraction of the reflected energy was directed towards the second mirror to, in sequence, fall directly on the sensor where the data will finally be processed. Figure 2a,b show the simplified scheme of the path of the radiative beam passing through the sample for measuring the directional/normal transmittance and the directional/normal reflectance, respectively. [9]

**Figure 3:**
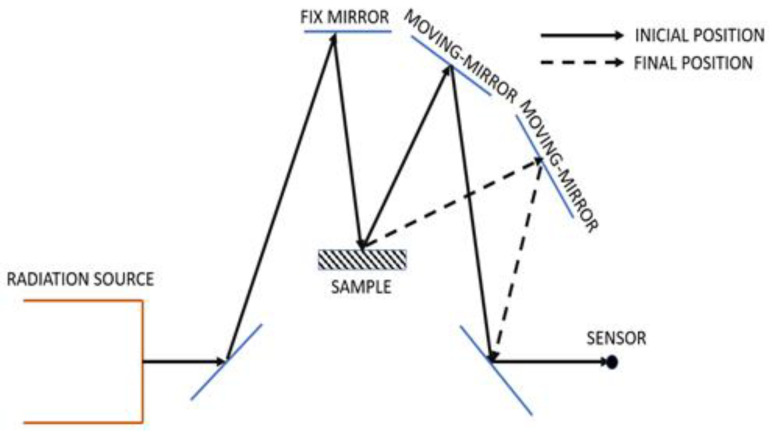
Diagram of the path traveled by a beam in a diffuse condition.

One of the mirrors was fixed for the diffuse reflectance measurement while the other was manually moved, one degree at time, within the lower and upper limits between the 10° and 35° interval; above this angle, the diffusely reflected signal was no longer detected, Figure 3.

**Figure 4:**
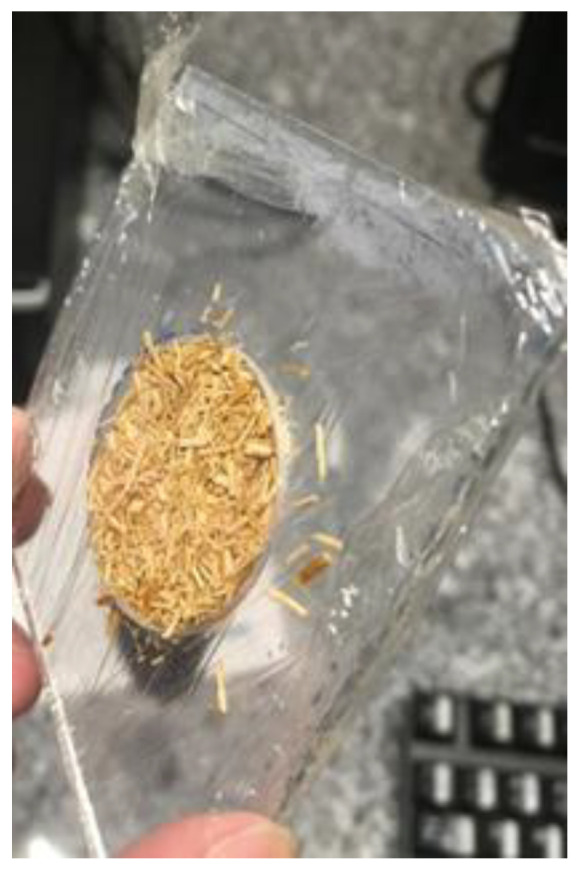
*Juncus maritimus* fiber placed in spectrometer sample holder apparatus, 30 mm in diameter.

To confine the fiber, which is a granulated material, in the 1 mm thick sample holder, a PVC plastic film was used, as shown in Figure 4. The plastic film acts in a participatory way, interfering in the results obtained in the fiber experiment [10].

**Figure 5:**
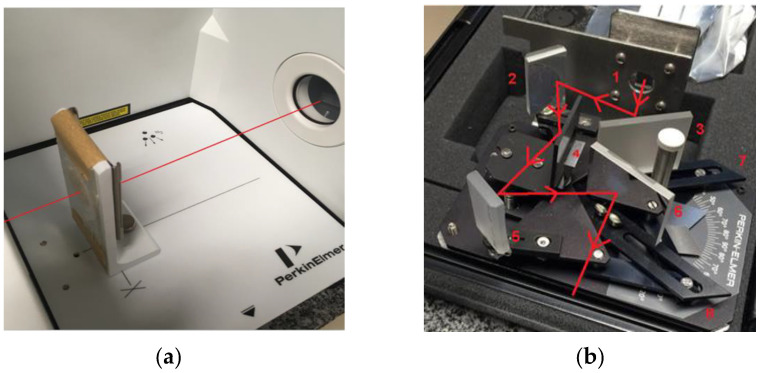
Diagram of the sample positioning inside the equipment for transmission (**a**) and reflection (**b**) measurements.

Figure 5a,b show how the samples were positioned inside the equipment before executing the normal and diffuse transmissibility and reflectivity tests, respectively. Numbers 2, 5, 3 and 6 in Figure 5b correspond to the moving mirrors of the accessory used for the reflection tests. Number 4 represents the sample positioned, while numbers 6, 7 and 8 indicate the mirror angle in meters. The opening indicated by the number 1 is from where the radiative beam leaves the FTIR to enter the reflection test accessory.

## 3. Discrete Ordinate Method

The Radiative Transfer Equation (RTE) was the mathematical equation used to describe the behavior of the radiation distribution within the material. The radiation propagates inside the fiber, interacts with the particulate, which partially absorbs the incident energy, transmitting and scattering the remaining amount. In addition to the energy carried by the external radiative beam, the same particulate can receive energy from its surroundings through thermal radiation from the medium itself. Neighborhood scattering can mitigate the intensity of the energy transmitted by the particulate, as part of the previously scattered intensity returns to the particulate in the form of reflected energy [11]. The radiation that returns to the particulate due to scattering from the surroundings, in a given direction, *μ*′, can be deflected to another direction, *μ*, which is numerically calculated by the phase function. The amount absorbed, transmitted and scattered strongly depends on the radiative parameters known as albedo *ω* and optical thickness *τ*, whose numerical values vary according to the wavelength, *λ*, of the incident radiative beam. To solve the problem, the following simplifications were considered [12]:

The sample is at room temperature, so the spectral band of the beam intensity emitted by the sample is different from the FTIR source.

The radiation propagates isotropically, with no preferred direction, through the material. As the particulate is scattered randomly, the material can also be considered homogeneous. The incident radiative beam fully spreads through the entire sample.

As previously discussed, the transfer equation depends on the phase function, $P_{\lambda }$ which determines the relationship between the intensity of an incident radiative beam on the particulate in the direction *μ*′, and the intensity of the radiative beam now emitted by the same particulate in the *μ* direction. Therefore, considering the conservation of energy, and the directional character of the beam, the RTE is mathematically expressed as [13]:

(1) $$\mu \frac{dI_{\lambda }}{dy}+\beta_{\lambda }I_{\lambda }=\frac{\sigma_{\lambda }}{2}\int_{-1}^{1}I_{\lambda }\left(y,\mu^{\prime }\right)P_{\lambda }\left(\mu^{\prime },\mu \right)d\mu^{\prime }$$

where: 

$I_{\lambda }$—Spectral intensity of radiation W·m^−2^·sr^−1^·μm^−1^

$\beta_{\lambda }$—Spectral volumetric extinction coefficient m^−1^

μ—Cosine of the scattered beam angle [-]

$\mu^{\prime }$—Cosine of the incident beam angle [-]

$P_{\lambda }$—Phase Function [-]

$\sigma_{\lambda }$—Spectral volumetric scattering coefficient m^−1^

**Figure 6:**
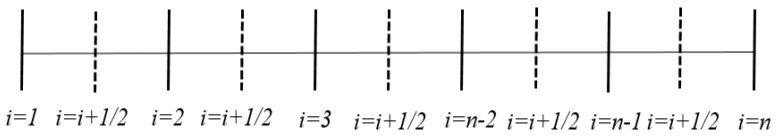
Volume discretization.

The spectral albedo, *ω*_l_, and the spectral optical thickness, *τ*_l_, are, respectively, defined as: $\sigma_{\lambda }/\beta_{\lambda }$ and $\beta_{\lambda }.l$ where l is the measure of the sample thickness in meters [m]. Equation (1) was rewritten in discrete ordinates and in a finite volume method for the implementation of the computational code. Both the term *S* and the intensity of the radiative beam $I_{\lambda }$, emitted in any direction *j*, are evaluated at the points indicated by the index *i*. *S* corresponds to the intensity of energy that affects the particulate due to the participation of the neighborhood on the volume and *I*. It is indicated the location of the central point of the differential volume. Figure 6 represents how the mesh was reproduced in a simplified way [14].

Equation (1) in discrete ordinates therefore becomes:

(2) $$I_{i+1/2,j}\;=\;\frac{S_{i+1/2,j}\alpha_{j}/2\;\;+\;I_{i,j}}{1\;+\;\alpha_{j}/2}$$

where:

(3) $$\alpha_{j}=\frac{\Delta \tau_{i+1/2}}{\mu_{j}}$$

(4) $$S_{i+1/2,j}=\frac{\omega }{2\beta }\sum_{n=1}^{N/2}w_{n}\left(P_{nj}I_{i+1/2,n}+P_{-nj}I_{i+1/2,-n}\right)$$

The variables $w_{n}$ and $P_{nj}$ correspond to the *n*-weights and phase function evaluated for the *n*-directions of the incident beams, used by the Gaussian quadrature, which numerically calculates the integral [15]. 

The boundary conditions for all experiments performed, normal transmittance and normal and diffuse reflectivity, are expressed as [13]:

(5) $$I\left(y=0,\mu \right)=\left\{\begin{array}{c} I_{0\lambda \;}\;\;\;\mu_{0}≅1\\ 0\;\;0<\mu <1 \end{array}\right.$$

$$I\left(y=l,\mu \right)=0$$

where:

l—Sample thickness [m]

$I_{0\lambda \;}$—The intensity of the collimated incident beam normally incident onto the sample [W·m^−2^sr^−1^μm^−1^]

The energy intensity was obtained through the following specified conditions:$\;I\left(y=l,\mu ≅1\right)$. The normal and diffuse reflected intensity was obtained following other conditions: $I\left(y=0,\mu ≅-1\right)$ and $\sum_{-1}^{0}I\left(y=0,-1<\mu \leq 0\right)$.

The value of the expected theoretical radiative intensities can be determined as a function of the parameters present in the RTE using the Discrete Ordinate Method. The determination of the parameters present in the RTE, as a function of the experimental data, will be presented in the next section.

## 4. Gaussian Linearization Method

As previously mentioned, the Gaussian linearization method was used to determine the physical parameters known as albedo and optical thickness that are indirectly present in the ETR, Equation (1). The expected theoretical values, which refer to the type of test performed, are calculated through the Taylor series expansion, which is expressed as follows:

(6) $$I_{ti}{\left.\left(p_{1}\ldots \;\ldots \;.,p_{n}\right)\right|}_{t=1,2,3}={\left.I_{ti}\right|}_{initial\;value}+\sum_{i=1}^{N=2}\frac{\partial I_{t}}{\partial p_{i}}\Delta p_{i}$$

The index *i* = 1 unto *N* = 2 indicates the parameters to be identified, which for this article correspond to the numerical values of albedo and optical thickness, respectively. In addition, the index *t* = 1, 2, 3 indicates the data obtained both theoretically and experimentally, depending on the type of experiment performed, which are the normal transmittance and the normal and diffuse reflectance, respectively.

The expansion of the Taylor series together with the Newton–Raphson method, which forces but does not guarantee the convergence between the theoretical values of the experimental values, adapted to the least squares method, generates the Gaussian linearization method. The minimization of the sum of squared error function is expressed mathematically as:

(7) $$\frac{\partial F_{ti}\left(p_{1}\ldots \ldots .,p_{n}\right)}{\partial p_{i}}=\frac{\partial }{\partial p_{i}}\sum_{t=1}^{N}{\left(I_{ti}-I_{ei}\right)}^{2}=0$$

Replacing Equation (6) into Equation (7), and expanding the derivatives of the sum of the squared error function $F_{ti}\left(p_{1}\ldots \ldots .,p_{n}\right)$ with respect to all dependent variables $p_{i}$ generates a set of equations that can be represented in matrix form as [16]:

(8) $$\left[\begin{array}{cc} \sum_{t=1}^{N}{\left(\frac{\partial I_{t}}{\partial p_{1}}\right)}^{2} & \sum_{t=1}^{N}\frac{\partial I_{t}}{\partial p_{1}}\frac{\partial I_{t}}{\partial p_{2}}\\ \sum_{t=1}^{N}\frac{\partial I_{t}}{\partial p_{2}}\frac{\partial I_{t}}{\partial p_{1}} & \sum_{t=1}^{N}{\left(\frac{\partial I_{t}}{\partial p_{2}}\right)}^{2} \end{array}\right]\left[\begin{array}{c} \Delta p_{1}\\ \Delta p_{2} \end{array}\right]=\left[\begin{array}{c} \sum_{t=1}^{N}\left(I_{ti}-I_{ei}\right)\frac{\partial I_{t}}{\partial p_{1}}\\ \sum_{t=1}^{N}\left(I_{ti}-I_{ei}\right)\frac{\partial I_{t}}{\partial p_{2}} \end{array}\right]$$

Therefore, the method consists of recursively solving the system generated by Equation (6) represented term-by-term in the following form, **A.X = B**, where: **A** indicates the sensitivity matrix, **X** the difference between the numerical values of the parameters to be determined from the values obtained by the previous iteration, and, finally, the source term **B,** which corrects the numerical values of the radiative beam intensities, depending on the type of experiment, calculated from the parameters obtained by the previous iterative method in relation to the experimental data. The initial values of the parameters to be determined can be considered the same for a material that has similar physical characteristics to the sample. In addition, the stopping condition of the recursive process depends on the tolerance that falls on the elements that make up the **X** matrix. 

In the next section, the results obtained by the computer simulation will be presented together with the experimental values of the tests performed.

## 5. Numerical Results

**Figure 7:**
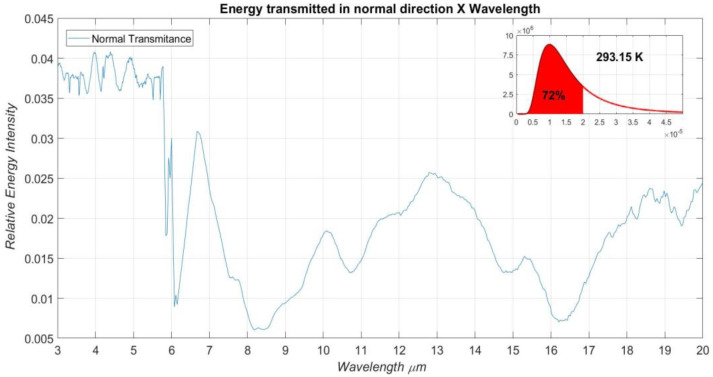
Relative intensity of the beam transmitted by the incident beam in the considered spectral band.

Figure 7 presents the experimental data of the beam intensity that crosses the sample, normalized in relation to the background signal, referring to the transmission measurement. Its assembly scheme is shown in Figure 5a. These measurements were performed for a spectral band that has wavelength values varying from 3 µm to 20 µm, which correspond to the energy emitted by the FTIR thermal source. The maximum value for the normalized intensity of the beam passing through the sample is 0.0408, and it occurs at a wavelength of approximately 3.928 µm. All theoretical data were treated considering the absorptivity of the plastic film [10].

**Figure 8:**
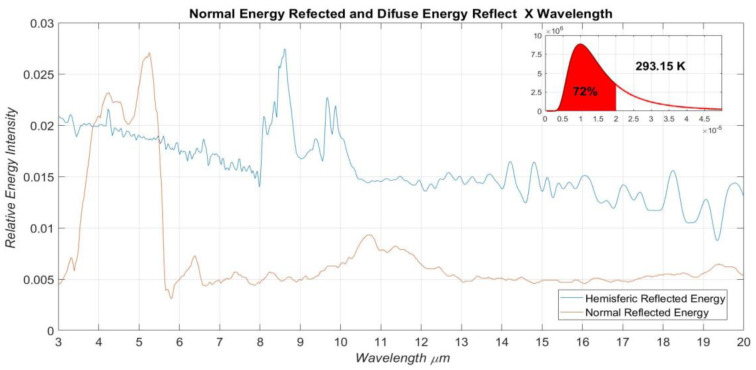
Specular and diffuse relative intensity of the beam reflected.

Figure 8 presents the experimental data related to the normal and diffuse reflectance measurements as a function of the same wavelength range of the normal transmittance measurement. The maximum relative values of the normally and diffusely reflected energy intensities are 0.0271 and 0.0268, respectively, which happens at the wavelengths of approximately 8.06 µm and 5.26 µm. The reflectance measurements were based on the assembly presented in Figure 2b and Figure 3. These experiments were conducted in the accessory pertinent to the reflectivity test, as shown in Figure 5b.

**Figure 9:**
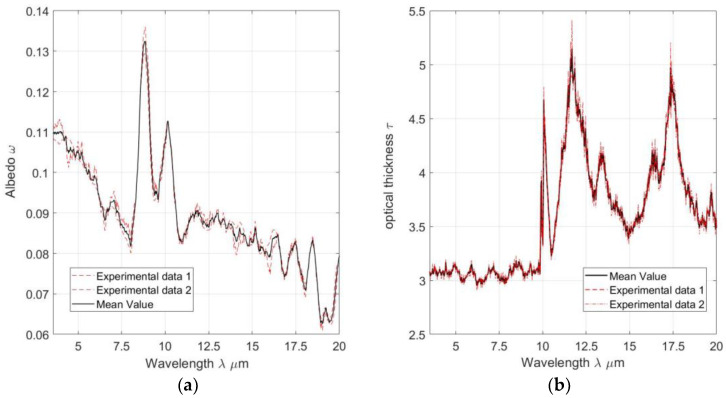
Data obtained from numerical simulation: (**a**) albedo found along the corresponding spectral band and (**b**) the optical thickness found along the same spectral band.

Figure 9a,b show, respectively: the albedo and optical thickness values. These were numerically determined by the Gaussian linearization method, also known as the inverse method, based on experimental data for the spectral band in which the experiment was carried out.

Through the volumetric extinction coefficient, $\beta_{\lambda }$, it is possible to determine the radiative conductivity, $K_{r}$, present in the Rosseland diffusion equation. Radiative conductivity is mathematically defined as [17]:

(9) $$K_{r}=\frac{3}{16\beta_{r}}\sigma T^{3}$$

Being: 

$K_{r}$—Radiative conductivity [W·m^−1^K^−1^]

$\beta_{r}$—Average Rosseland attenuation coefficient [m^−1^]

σ—Stefan–Boltzmann constant [W·m^−2^K^−4^]

*T*—Temperature [K]

For this work, the Stefan–Boltzmann constant was numerically considered as 5.6705119 × 10^−8^ [W·m^−2^K^−4^], and the Rosseland mean attenuation coefficient, defined as [17]:

(10) $$\frac{1}{\beta_{r}}=\int_{\lambda_{1}}^{\lambda_{2}}\frac{1}{\beta \left(\lambda \right)}\frac{\pi }{2}\frac{C_{1}C_{2}}{\lambda^{6}}\frac{e^{\left(C_{2}/\lambda T\right)}}{{\left[e^{\left(C_{2}/\lambda T\right)}-1\right]}^{2}}\frac{\sigma^{1/4}}{B^{5/4}}d\lambda /\int_{\lambda_{1}}^{\lambda_{2}}\frac{\pi }{2}\frac{C_{1}C_{2}}{\lambda^{6}}\frac{e^{\left(C_{2}/\lambda T\right)}}{{\left[e^{\left(C_{2}/\lambda T\right)}-1\right]}^{2}}\frac{\sigma^{1/4}}{B^{5/4}}d\lambda$$

where: 

$C_{1}$ = 0.59544 × 10^−16^ [W·m^2^]

$C_{2}$ = 14,388 × 10^−6^ [m·K]

Applied to the wavelength range between $\lambda_{1}\;\mathrm{and}\;\lambda_{2}$ defined from 3 µm to 20 µm, and considering $B=\sigma T^{4}$, the radiative conductivity of the *Juncus maritimus* fiber obtained was $K_{r}$, 0.0022 [W·m^−1^K^−1^]. 

The condition number is the tool needed to determine if the problem converges. An ill-conditioned matrix means that a minor change in the source term, represented by Vector B of Equation (6), corresponds to a very large change in vector X. According to [18], a matrix with the condition number below 20 is considered an already well-conditioned matrix. The conditioning number is defined as:

(11) $$Condition\;number=\|A\|\left\|A^{-1}\right\|$$

where: 

‖A‖—Matrix A norm

$\left\|A^{-1}\right\|$—Matrix A inverse norm

For this work, the matrix norm is defined as:

(12) $$\|A_{m\;x\;n}\|={\left(\sum_{i=1}^{m}\sum_{j=1}^{n}{a_{ij}}^{2}\right)}^{\frac{1}{2}}$$

**Figure 10:**
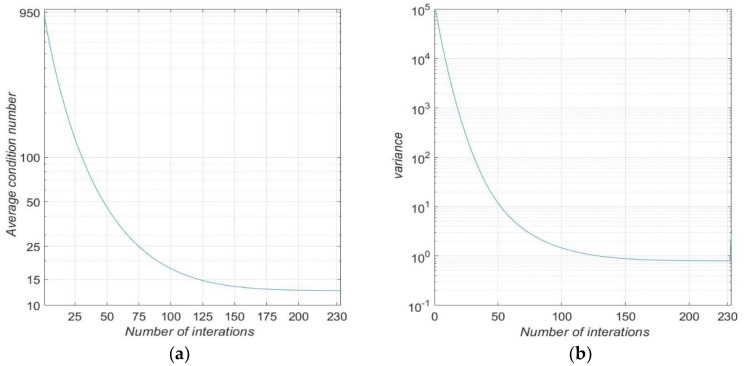
Conditioning number of matrices. (**a**) Average conditioning number of matrices for each average wavelength as a function of iterations and (**b**) variation of conditioning function number for each of the iterations.

Figure 10a presents the average conditioning number, considering the number of iterations performed for each wavelength. Figure 10b also presents the variance corresponding to the results obtained in Figure 10a. The variance of the matrix conditioning value, for the last iterations, shows that the system is consistent for the entire wavelength range considered. The values found for the parameters are successively reused for the next iterative process corresponding to a new wavelength. Therefore, for values in which the experimental data of the neighborhood are similar to each other, the problem converges faster. In addition, this type of behavior justifies the dispersion of variance at the beginning of the process, as shown in Figure 10b.

**Figure 11:**
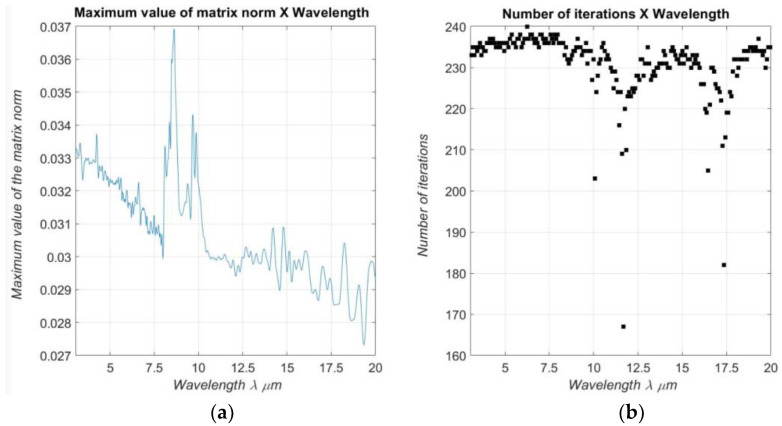
Matrix Norm: (**a**) Maximum values of the matrix norm corresponding to each simulation for each corresponding wavelength. (**b**) Number of iterations performed for each wavelength.

As proposed, the present work uses the definition of the Neumann series as an alternative to find the solution for the linear problem, **A.X = B,** which is generated for each iteration. The Neumann series is defined as:$\;A^{-1}\approx \sum_{i}^{n}{\left(I-A\right)}^{n}$ where I is an identity matrix and the exponent *n* the series expansion order, which is a variable strongly dependent on the precision required by the problem [6]. The Neumann series is applicable to any problem if, and only if, ‖A‖<1 [18,19]. Figure 11a,b show the maximum value for the norm of the sensitivity matrix A for each wavelength and the number of iterations also performed in wavelength function, respectively.

**Figure 12:**
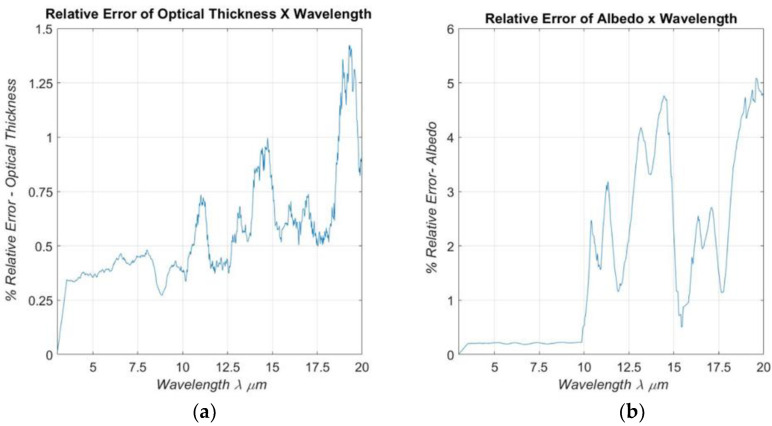
Relative distance: (**a**) spectral optical thickness and (**b**) spectral albedo calculated by the Neumann method compared with the Gauss–Jordan method for all corresponding wavelengths.

Figure 12a,b present the relative percentage deviation of albedo and optical thickness, respectively, calculated using the Neumann method in relation to the Gauss–Jordan method. The deviation was obtained through the mathematical relation: $\left|X_{n}-X_{g}\right|/X_{g}$, where $X_{n}$ indicates the parameter value obtained through the Neumann method and $X_{g}$ is the parameter value obtained through the Gauss method.

The computational cost of the Neumann and Gauss method were, respectively, 5.6782 × 10^3^ s and 4.9907 × 10^3^ s, the difference between the values was small because the sensitivity matrix is of order 2. The system solution tolerance represented by the equation: **A·X = B**, using the Neumann method for this work, was considered in the order of 10^−5^. For higher order sensitivity matrices, where we aim to identify additional parameters, and for a lower level of demand, the Neumann method becomes significantly more advantageous than other traditional methods [20].

## 6. Conclusions

As shown in this work, it is possible to mathematically determine the radiative properties of *Juncus maritimus* found in Tunisia, mainly the albedo and optical thickness, through the Gaussian linearization method, which uses the values generated by the experiments performed. According to what has been presented, the fiber absorbs most of the incident energy, something that was already expected in this work.

The contribution of this work is not restricted only to the determination of the radiative parameters of the fiber, but also to the use of the Neumann method for the solution of the linear system, at each iteration. The Neumann method can represent an economical computational time if the idea is to determine a larger number of parameters, which increases the order of the sensitivity matrix when compared to other methods for solving linear systems—Gauss–Jordan, LU decomposition, etc.

Another fundamental contribution of this work was finding the radiative conductivity of the *Juncus maritimus* fiber for the wavelength range which corresponds to the region where the energy intensity emitted is most prominent at room temperature. The radiative conductivity allows further simplifications for finding the global conductivity that corresponds to the flux contribution of the energy intensity, both from the conduction and the radiation, especially for porous media. It is important to mention that, as expected, the junco fiber proved to be an organic material that absorbs practically all energy in the form of radiation for the considered spectrum.
